# High genetic diversity in *Campylobacter concisus* isolates from patients with microscopic colitis

**DOI:** 10.1186/s13099-020-00397-y

**Published:** 2021-01-12

**Authors:** Marta Emilie Yde Aagaard, Karina Frahm Kirk, Henrik Nielsen, Hans Linde Nielsen

**Affiliations:** 1 Department of Infectious Diseases, Aalborg University Hospital, Hobrovej 18-22, 9000 Aalborg, Denmark; 2 Department of Clinical Medicine, Aalborg University, Søndre Skovvej 15, 9000 Aalborg, Denmark; 3 Department of Clinical Microbiology, Aalborg University Hospital, Hobrovej 18-22, 9000 Aalborg, Denmark

**Keywords:** *Campylobacter concisus*, Microscopic colitis, Genetic diversity, Multi-locus sequence typing, Phylogenetic relatedness, Inflammatory bowel disease, Genomospecies

## Abstract

The emerging intestinal pathogen *Campylobacter concisus* has been associated with prolonged diarrhoea and classic inflammatory bowel diseases (IBD) and was recently also linked with microscopic colitis (MC). Previous reports have observed a high genetic diversity within isolates from diarrhoeic and IBD patients and from healthy controls (HC), and division of isolates into two major genomospecies (GS1 and GS2). The aim of this study was to describe genetic diversity in 80 recently cultivated MC biopsy and faecal isolates of *C. concisus* by multi-locus sequence typing (MLST); and to compare the phylogenetic relatedness to 102 isolates from diarrhoeic and IBD patients and HCs by k-mer-based distance estimation. MLST revealed high genetic diversity in MC isolates with 72 novel sequence types. K-mer divided MC isolates into two distinct clusters (cluster 1 n = 21, cluster 2 n = 49), with a significantly higher prevalence of cluster 2 isolates in biopsies than in faeces, p = 0.009. K-mer divided the 182 isolates into two major phylogenetic clusters: cluster 1 (GS1 isolates) and cluster 2 (GS2 isolates), which further differentiated into three subgroups. Cluster 1 and the three cluster 2 subgroups were each distinctive in mean genome size and GC count. Isolates from all disease phenotypes were present in cluster 1 and cluster 2 subgroup 2 and 3, whereas cluster 2 subgroup 1 only contained isolates restricted to patients with ulcerative colitis (n = 10) and HC (n = 4).

## Introduction

The *Campylobacter* genus encompasses 33 species and 8 subspecies [1], which cluster into five phylogenetic groups, all containing pathogenic species capable of causing human infection [2]. *Campylobacter concisus* was first reported from human periodontal lesions [3] and the human oral cavity is now acknowledged as its natural colonisation site [2, 4]. *C. concisus* has been considered an emerging intestinal pathogen associated with prolonged diarrhoea, ulcerative colitis (UC) and Crohn’s disease (CD) [4–7]. However, *C. concisus* has also been isolated in high numbers from healthy controls (HC) [2, 4, 5].

Microscopic colitis (MC) is an inflammatory bowel disease of the colon that primarily affects post-menopausal women [8]. It encompasses the two subtypes collagenous colitis (CC) and lymphocytic colitis (LC), which both cause watery diarrhoea [8]. The aetiology and pathogenesis of MC remains unclear, but luminal gut factors are hypothesised to play a part in onset and maintenance of the chronic condition [8, 9]. A recent population-based cohort study showed an increased risk of MC after *C. concisus* in stools and the hazard ratio was almost twice as high than observed in patients with culture-negative stools [10]*.*

Studies have shown that *C. concisus* strains can be divided into two main genomospecies (GS1 and GS2), primarily based on amplified fragment length polymorphism (AFLP) [11–13] and 23S rRNA analysis [14–18]. Interestingly, GS2 isolates have larger genome sizes and consist of more genes per genome than GS1 isolates; and GS2 isolates predominate mucosal biopsy isolates, whereas GS1 isolates predominate oral samples [18]. However, differentiation into GS1 and GS2 has not been linked to disease phenotype or pathogenicity [18]. Furthermore, multi-locus sequence typing (MLST) has shown that strains of *C. concisus* from diarrheic patients, UC and CD patients and HC are highly diverse with a very high number of sequence types (STs) [15, 18, 19]. Whether these highly genomic differences in *C. concisus* strains are related to possible pathogenic differences remain to be established.

We recently reported a high number of *C. concisus* isolates cultivated from faeces and colonic mucosal biopsies from MC patients [17]. Isolates were sequenced by whole-genome shotgun sequencing (WGS) and the GS distribution and prevalence of putative virulence genes (*zot*, Exotoxin-9 and *hcp*) was reported [17]. Our aim with this study was to describe genetic diversity in *C. concisus* strains isolated from MC patients by MLST analysis and k-mer distance estimation; and to compare phylogenetic relatedness to previously sequenced and public available UC, CD, diarrhoeal and HC *C. concisus* genomes from our group.

## Methods

Eighty *C. concisus* isolates (60 biopsy and 20 faecal) collected from 19 patients with MC (9 females and 10 males) were analysed. Patients presented as 14 CC and 5 LC patients with a mean age of 66 years (range 37–88). All isolates were collected and sequenced as previously described [17]. In brief, DNA was extracted using the DNeasy^®^ Ultraclean^®^ Microbial Kit (QIAGEN, Hilden, Germany). Nextera XT DNA Library Prep Kit was used for library preparation and next generation sequencing was performed by use of the Illumina Miseq platform (Illumina, San Diego, USA). Generated FASTQ files were trimmed and assembled within the CLC Genomics Workbench 12.0.3 (QIAGEN, Hilden, Germany) with use of *C. concisus* ATCC 33237, 13826 and P2CD04 as reference genomes.

Analysis of sequence types and housekeeping loci was performed with generated FASTA files by use of the Miller MLST scheme, which is based on the seven housekeeping genes: *aspA*, *atpA*, *glnA*, *gltA*, *glyA*, *ilvD* and *pgm* [15]. MLST results were generated by use of the online web tool MLST 2.0, using the *Campylobacter concisus/curvus* configuration [20, 21]. Sequences of the seven housekeeping genes in each *C. concisus* genome were manually aligned with every other genome of the 80 MC isolates.

Furthermore, phylogenetic trees were constructed by k-mer distance estimation with standard parameters (both strands, k-mer length 16, ATGAC as prefix and with Feature frequency profile-based calculation) within the microbial genomics module of the CLC Genomics Workbench 12.0.3. MC isolates were analysed with regard to site of isolation (faeces or biopsy) and MC subtype (CC or LC), and further compared with a total of 100 assembled genomes from Kirk et al. [18] (NCBI Bio-project accession: PRJNA395841) and the complete genomes of *C. concisus* ATCC 33237 and 13826.

Fisher’s exact test was used for dichotomous variables in StataMP 16 (Statacorp LP, Texas, USA). A p-value < 0.05 was considered statistically significant.

## Results

MLST analysis revealed a high number of alleles in our collection of MC isolates, leading to a very high genetic diversity with 72 novel STs (Table 1). Additional file 1 presents the full list of MC *C. concisus* isolates used in this study. Interestingly, one patient was colonised with the same ST in all four biopsy isolates from different locations, whereas remaining patients with multiple biopsy isolates presented with at least three different STs (Additional file 1: Table S1).

**Table 1:** Sequence types (STs) and alleles identified by the Miller MLST scheme

Strains (n)	STs	Alleles^a^						
		*aspA*	*atpA*	*glnA*	*gltA*	*glyA*	*ilvD*	*pgm*
80	72	42	39	45	47	38	39	43

See main text for reference.

^a^The allelic numbers refers to the total number of different alleles within each housekeeping gene in the 80 *C. concisus* strains.

**Fig. 1:**
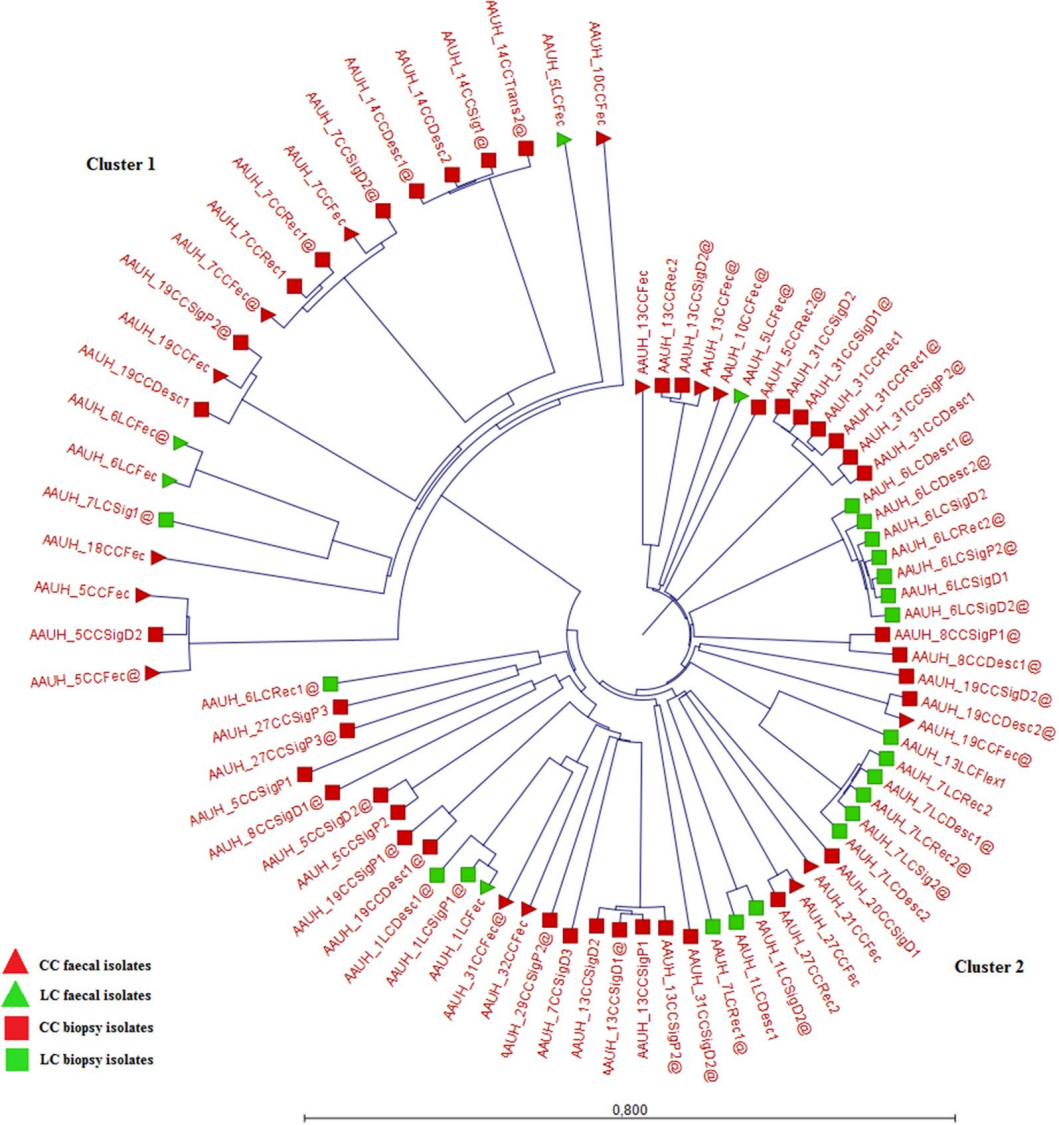
Circular tree based on k-mer distance estimation of MC isolates revealed two distinct clusters with cluster 1 (GS1) (n = 21) and cluster 2 (GS2) (n = 59). Twenty faecal isolates (triangles) were divided equally into cluster 1 and 2 (n = 10 in each cluster), which was significantly different from the 60 biopsy isolates (squares) that mainly belonged to cluster 2 (n = 49). No significant differences in cluster differentiation were observed in isolates from CC patients (red colour) (cluster 1 n = 17 and cluster 2 n = 38) and from LC patients (green colour) (cluster 1 n = 4 and cluster 2 n = 21)

Cluster differentiation by k-mer distance estimation revealed two distinct clusters when analysing MC *C. concisus* isolates (Fig. 1). Faecal isolates divided equally into cluster 1 and 2 (n = 10 in each cluster), which was significantly different from biopsy isolates that were predominated by cluster 2 isolates (n = 49) (cluster 1, n = 11), p = 0.009. No differences were observed, when analysing isolates regarding MC subtypes: CC isolates (cluster 1 n = 17, cluster 2 n = 38), LC isolates (cluster 1 n = 4, cluster 2 n = 21), p = 0.18.

When comparing phylogenetic relatedness between multiple *C. concisus* strains, k-mer distance estimation revealed differentiation into two main clusters (Additional file 2: Figure S1). Cluster 1 (n = 52) consisted of strains previously identified as GS1 [17, 18] and presented with a mean genome size of 1.91 Mbp (range 1.78–2.33) and a GC count of 37.6% (range 36.4–37.9). Cluster 2 strains were all isolates previously identified as GS2 [17, 18], but interestingly this cluster differentiated into three different subgroups based on phylogenetic distance estimation. Cluster 2 subgroup 1 strains (n = 14) were closely phylogenetically related to cluster 1 isolates but presented with a mean genome size of 2.01 Mbp (range 1.94–2.08) and a GC count of 39.6% (range 39.4–39.8). Cluster 2 subgroup 2 isolates (n = 50) had the largest mean genome size of 2.10 Mbp (range 1.91–2.30) and a GC count of 39.4% (range 38.9–40.1). Finally, cluster 2 subgroup 3 isolates (n = 64) presented with a mean genome size of 1.96 Mbp (range 1.81–2.20) and a GC count of 39.5% (range 39.1–39.8). The reference genomes were present in cluster 1 (ATCC 33237) and cluster 2 subgroup 1 (*C. concisus* 13826) and were not included in the analysis of genome size or GC count. All clusters and subgroups presented with isolates from all disease phenotypes, except for cluster 2 subgroup 1, which only contained 10 UC and 4 HC isolates and the reference genome *C. concisus* 13826.

## Discussion

In this study, we present results from MLST analysis of 80 *C. concisus* isolates from Danish MC patients. We observed that MC isolates were highly genetic diverse, which is in concordance with previous observations on *C. concisus* isolates from patients with inflammatory bowel disease (UC or CD) or prolonged diarrhoea and from HC [15, 18, 19]. Furthermore, phylogenetic k-mer distance estimation demonstrated that MC isolates cluster into two distinct clusters. This cluster differentiation is identical with the GS distribution previously observed by 23S rRNA, Average Nucleotide Identity and Genome BLAST distance phylogeny analysis as previously reported [17]. *Campylobacter concisus* differentiation into distinct clusters has been hypothesised to explain differences in pathogenic potential among *C. concisus* strains. Kalischuk et al. [12] reported that AFLP cluster 2 isolates had higher epithelial invasion and translocation rates in human T84 epithelial cells than AFLP cluster 1 isolates, suggesting higher pathogenic potential in cluster 2. However, AFLP cluster 1 isolates stimulated a higher IL-8 mRNA expression compared with cells infected with AFLP cluster 2 isolates [12]. In addition, both AFLP 1 (n = 1) [13] and AFLP 2 (n = 4) [11] strains have been associated with bloody diarrhoea. AFLP clustering is to some extent identical to GS differentiation [12, 15], however the distribution of isolates into clusters may vary with regard to the methodological approach. Furthermore, Kirk et al. [18] reported that differentiation into genomospecies was not related to disease phenotype, as isolates from HC and patients with UC, CD and diarrhoea are present in both GS1 and GS2. However, GS differentiation did appear to be associated with the site of sample collection, as GS1 isolates are more prevalent in oral samples and GS2 isolates in intestinal biopsy samples, whereas faecal isolates divide equally into GS1 and GS2 [17–19]. GS distribution may therefore not be related to pathogenicity of *C. concisus* isolates but adaptation to different colonisation sites. Nevertheless, further studies into immunological and physiological effects of GS1 and GS2 isolates in intestinal cell lines would be of interest to elucidate possible pathogenic differences between genomospecies.

Interestingly, when comparing phylogenetic relatedness among *C. concisus* genomes from patients with MC, UC, CD and diarrhoea and from HC, several subgroups within cluster 2 appeared in the present study. The genomes clustered into one GS1 cluster based on genome size and GC count and the three subgroups in cluster 2 consisted of GS2 isolates [17, 18]. Fifty-seven faecal and five oral *C. concisus* strains were previously reported to cluster into 4 distinct clusters based on AFLP analysis [11]. However, the single isolate in cluster 3 (GS3) was later placed in GS2 by Miller et al. [15], and the five isolates in cluster 4 (GS4) did not yield an amplicon in 23S rRNA analysis and were later re-identified as *Campylobacter curvus* isolates by MLST [11, 15]. In addition, another study revealed two novel genomospecies (GS5 and GS6) based on AFLP analysis, each containing one isolate (GS5: Lasto 127.99 and GS6: Lasto 393.96) [13]. On et al. further analysed these isolates by 23S rRNA, which placed the isolates in GS2 [13]. However, by including the public available assembled genomes of these isolates into our k-mer based phylogenetic tree, Lasto 393.96 (genome size: 1.85 Mbp, GC count: 37.4%) was placed in cluster 1 and Lasto 127.99 (genome size: 2.03 Mbp, GC count: 39.4%) in cluster 2 subgroup 3 (data not shown) [22]. We did not observe distinct distribution of isolates from different disease phenotypes into the different clusters and cluster subgroups in the present study. The observed clustering may possibly be the result of differences in genome size and GC count among the isolates, but also hypothetically related to isolation from specific anatomic niches in which strains have optimal conditions of colonisation [18]. However, the numbers of oral isolates were too small for sufficient interpretation (n = 13), even though cluster 1 contained 11 of these isolates. Whether the observed clustering is of pathogenic importance remains to be explored.

In conclusion, MLST analysis revealed high genetic diversity in 80 MC *C. concisus* isolates. K-mer distance estimation demonstrated two distinct clusters in MC isolates, which were in concordance with previous GS1 and GS2 analysis. However, no difference was observed between CC and LC strains, and GS may therefore primarily be related to the anatomical niche in which *C. concisus* has been isolated. MC isolates were phylogenetically closely related to the genomes of *C. concisus* isolated from patients with UC, CD and diarrhoea and from HC.

## Supplementary Information

**Additional file 1: Table S1.** Presentation of each MC *C. concisus* genome used for MLST analysis and k-mer distance estimation (n = 80). Information includes clinical data, isolate origin, GS data and ST.

**Additional file 2: Figure S1.** Circular tree based on k-mer distance estimation revealed two distinct clusters: cluster 1 and cluster 2. Cluster 2 isolates further differentiated into three subgroups. Cluster 1 (from AAUH_7CCFec@ to AAUH_12CDo) contained 52 GS1 isolates and the reference genome ATCC 33237. Cluster 2 subgroup 1 (from AAUH_44sig@ to AAUH_20HCsig@) contained 14 GS2 isolates and the reference genome 13826. Cluster 2 subgroup 2 (from AAUH_31CCRec1@ to AAUH_40UCf) contained 50 GS2 isolates. Cluster 2 subgroup 3 (from AAUH_27CCSigP3@ to AAUH_16UCdp5) contained 64 GS2 isolates. Branches indicate origin of the isolates: MC (blue) and isolates from Kirk et al. [18] and the reference genomes (yellow).

## Data Availability

The datasets generated and/or analysed during the current study are not publicly available yet but are available from the corresponding author on reasonable request.

## Abbreviations

HC: Healthy controls
UC: Ulcerative colitis
CD: Crohn’s disease
MC: Microscopic colitis
AFLP: Amplified fragment length polymorphism
MLST: Multi-locus sequence typing
GS: Genomospecies
ST: Sequence type
CC: Collagenous colitis
LC: Lymphocytic colitis
