# Supplementary material for: High genetic diversity in Campylobacter concisus isolates from patients with microscopic colitis
Source: Gut Pathog. 2021 Jan 12;13:3. doi: 10.1186/s13099-020-00397-y (PMC7805038; doi:10.1186/s13099-020-00397-y)
Supplement: Supplementary file 1 — Additional file 1: Table S1. Presentation of each MC C. concisus genome used for MLST analysis and k-mer distance estimation (n = 80). Information includes clinical data, isolate origin, GS data and ST. [file 13099_2020_397_MOESM1_ESM.docx]

| Patient ID | Age | Sex | MC diagnosis | Isolate ID | Source | Genomospecies | Novel ST |
| --- | --- | --- | --- | --- | --- | --- | --- |
| 1 | 66 | Male | LC | AAUH_1LCDesc1 | Biopsy | 2 | 1 |
|  |  |  |  | AAUH_1LCDesc1@ (a) | Biopsy | 2 | 2 |
|  |  |  |  | AAUH_1LCSigP1@ (a) | Biopsy | 2 | 2 |
|  |  |  |  | AAUH_1LCSigD2@ | Biopsy | 2 | 3 |
|  |  |  |  | AAUH_1LCFec (a) | Faeces | 2 | 2 |
| 2 | 70 | Male | LC | AAUH_5LCFec | Faeces | 1 | 4 |
|  |  |  |  | AAUH_5LCFec@ | Faeces | 2 | 5 |
| 3 | 75 | Male | CC | AAUH_5CCSigP1 | Biopsy | 2 | 6 |
|  |  |  |  | AAUH_5CCSigP2 | Biopsy | 2 | 7 |
|  |  |  |  | AAUH_ 5CCSigD2 (b) | Biopsy | 1 | 8 |
|  |  |  |  | AAUH_5CCSigD2@ | Biopsy | 2 | 9 |
|  |  |  |  | AAUH_5CCRec2@ | Biopsy | 2 | 10 |
|  |  |  |  | AAUH_5CCFec (b) | Faeces | 1 | 8 |
|  |  |  |  | AAUH_5CCFec@ | Faeces | 1 | 11 |
| 4 | 56 | Female | LC | AAUH_6LCDesc1@ | Biopsy | 2 | 12 |
|  |  |  |  | AAUH_6LCDesc2@ | Biopsy | 2 | 13 |
|  |  |  |  | AAUH_6LCSigP2@ | Biopsy | 2 | 14 |
|  |  |  |  | AAUH_6LCSigD1 | Biopsy | 2 | 15 |
|  |  |  |  | AAUH_6LCSigD2 | Biopsy | 2 | 16 |
|  |  |  |  | AAUH_6LCSigD2@ | Biopsy | 2 | 17 |
|  |  |  |  | AAUH_6LCRec1@ | Biopsy | 2 | 18 |
|  |  |  |  | AAUH_6LCRec2@ | Biopsy | 2 | 19 |
|  |  |  |  | AAUH_6LCFec | Faeces | 1 | 20 |
|  |  |  |  | AAUH_6LCFec@ | Faeces | 1 | 21 |
| 5 | 67 | Female | LC | AAUH_7LCDesc1@ | Biopsy | 2 | 22 |
|  |  |  |  | AAUH_7LCDesc2 | Biopsy | 2 | 23 |
|  |  |  |  | AAUH_7LCSig1@ | Biopsy | 1 | 24 |
|  |  |  |  | AAUH_7LCSig2@ | Biopsy | 2 | 25 |
|  |  |  |  | AAUH_7LCRec1@ | Biopsy | 2 | 26 |
|  |  |  |  | AAUH_7LCRec2 | Biopsy | 2 | 27 |
|  |  |  |  | AAUH_7LCRec2@ | Biopsy | 2 | 28 |
| 6 | 65 | Female | CC | AAUH_7CCSigD2@ (c) | Biopsy | 1 | 29 |
|  |  |  |  | AAUH_7CCSigD3 | Biopsy | 2 | 30 |
|  |  |  |  | AAUH_7CCRec1 (c) | Biopsy | 1 | 29 |
|  |  |  |  | AAUH_7CCRec1@ | Biopsy | 1 | 31 |
|  |  |  |  | AAUH_7CCFec | Faeces | 1 | 32 |
|  |  |  |  | AAUH_7CCFec@ | Faeces | 1 | 33 |
| 7 | 88 | Male | CC | AAUH_8CCDesc1@ | Biopsy | 2 | 34 |
|  |  |  |  | AAUH_8CCSigP1@ | Biopsy | 2 | 35 |
|  |  |  |  | AAUH_8CCSigD1@ | Biopsy | 2 | 36 |
| 8 | 74 | Male | CC | AAUH_10CCFec | Faeces | 1 | 37 |
|  |  |  |  | AAUH_10CCFec@ | Faeces | 2 | 38 |
| 9 | 53 | Male | LC | AAUH_13LCFlex1 | Biopsy | 2 | 39 |
| 10 | 72 | Male | CC | AAUH_13CCSigP1 | Biopsy | 2 | 40 |
|  |  |  |  | AAUH_13CCSigP2@ | Biopsy | 2 | 41 |
|  |  |  |  | AAUH_13CCSigD1@ | Biopsy | 2 | 42 |
|  |  |  |  | AAUH_13CCSigD2 | Biopsy | 2 | 43 |
|  |  |  |  | AAUH_13CCSigD2@ | Biopsy | 2 | 44 |
|  |  |  |  | AAUH_13CCRec2 | Biopsy | 2 | 45 |
|  |  |  |  | AAUH_13CCFec | Faeces | 2 | 46 |
|  |  |  |  | AAUH_13CCFec@ | Faeces | 2 | 47 |
| 11 | 68 | Male | CC | AAUH_14CCTrans2@ (d) | Biopsy | 1 | 48 |
|  |  |  |  | AAUH_14CCDesc1@ (d) | Biopsy | 1 | 48 |
|  |  |  |  | AAUH_14CCDesc2 (d) | Biopsy | 1 | 48 |
|  |  |  |  | AAUH_14CCSig1@ (d) | Biopsy | 1 | 48 |
| 12 | 70 | Female | CC | AAUH_18CCFec | Faeces | 1 | 49 |
| 13 | 68 | Female | CC | AAUH_19CCDesc1 | Biopsy | 1 | 50 |
|  |  |  |  | AAUH_19CCDesc1@ | Biopsy | 2 | 51 |
|  |  |  |  | AAUH_19CCDesc2@ | Biopsy | 2 | 52 |
|  |  |  |  | AAUH_19CCSigP1@ | Biopsy | 2 | 53 |
|  |  |  |  | AAUH_19CCSigP2@ | Biopsy | 1 | 54 |
|  |  |  |  | AAUH_19CCSigD2@ | Biopsy | 2 | 55 |
|  |  |  |  | AAUH_19CCFec | Faeces | 1 | 56 |
|  |  |  |  | AAUH_19CCFec@ | Faeces | 2 | 57 |
| 14 | 66 | Male | CC | AAUH_20CCSigD1 | Biopsy | 2 | 58 |
| 15 | 88 | Female | CC | AAUH_21CCFec | Faeces | 2 | 59 |
| 16 | 65 | Male | CC | AAUH_27CCSigP3 | Biopsy | 2 | 60 |
|  |  |  |  | AAUH_27CCSigP3@ | Biopsy | 2 | 61 |
|  |  |  |  | AAUH_27CCRec2 | Biopsy | 2 | 62 |
|  |  |  |  | AAUH_27CCFec | Faeces | 2 | 63 |
| 17 | 70 | Female | CC | AAUH_29CCSigP2@ | Biopsy | 2 | 64 |
| 18 | 72 | Female | CC | AAUH_31CCDesc1 | Biopsy | 2 | 65 |
|  |  |  |  | AAUH_31CCSigP2@ | Biopsy | 2 | 66 |
|  |  |  |  | AAUH_31CCSigD1@ (e) | Biopsy | 2 | 67 |
|  |  |  |  | AAUH_31CCSigD2 | Biopsy | 2 | 68 |
|  |  |  |  | AAUH_31CCSigD2@ | Biopsy | 2 | 69 |
|  |  |  |  | AAUH_31CCRec1 (e) | Biopsy | 2 | 67 |
|  |  |  |  | AAUH_31CCRec1@ | Biopsy | 2 | 70 |
|  |  |  |  | AAUH_31CCFec@ | Faeces | 2 | 71 |
| 19 | 37 | Female | CC | AAUH_32CCFec | Faeces | 2 | 72 |
| * identical STs are marked with a, b, c, d or e | | |  |  |  |  |  |
