# Supplementary figures and images for: High genetic diversity in Campylobacter concisus isolates from patients with microscopic colitis
Source: Gut Pathog. 2021 Jan 12;13:3. doi: 10.1186/s13099-020-00397-y (PMC7805038; doi:10.1186/s13099-020-00397-y)

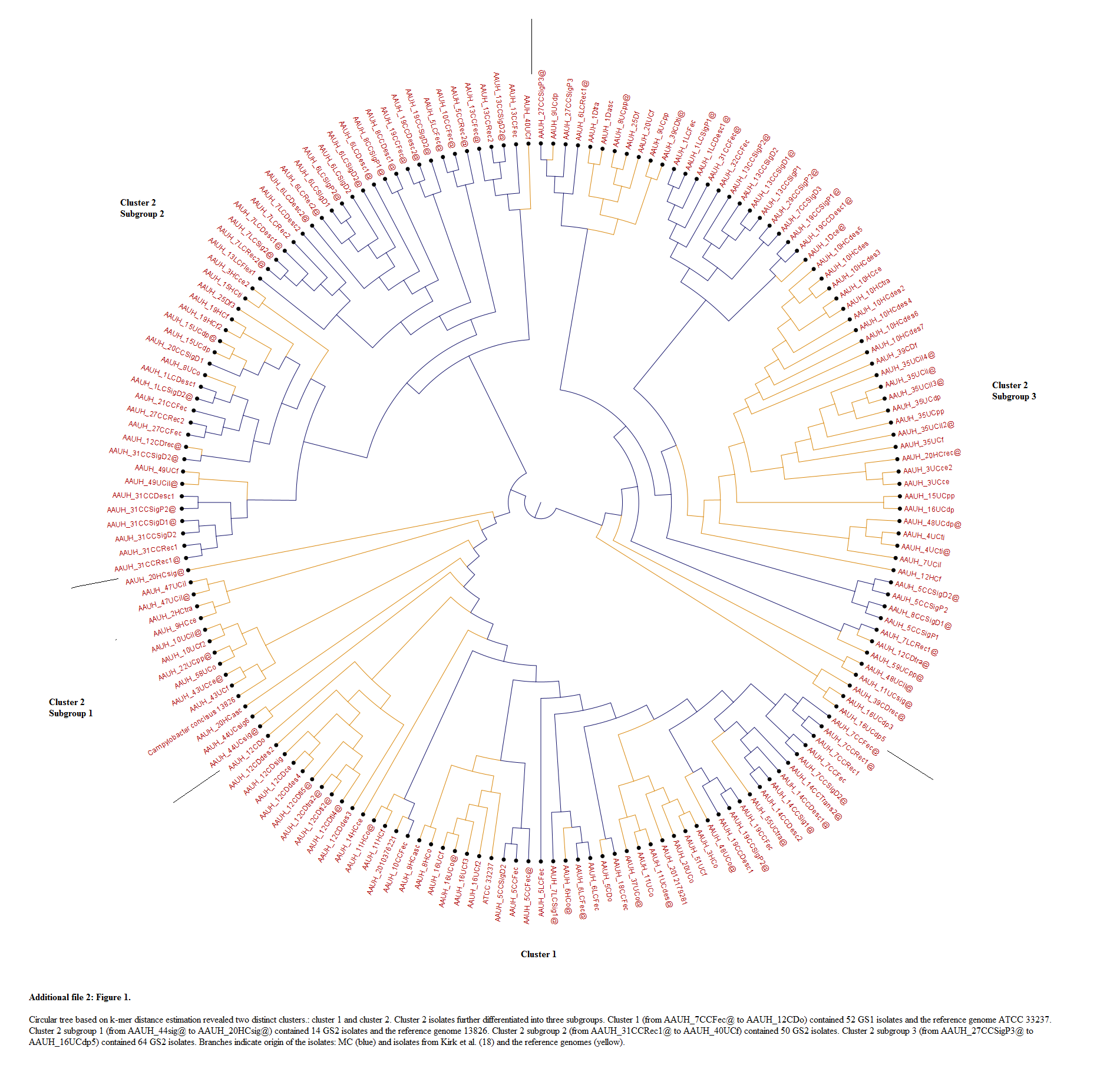

Supplement: Supplementary file 2 — Additional file 2: Figure S1. Circular tree based on k-mer distance estimation revealed two distinct clusters: cluster 1 and cluster 2. Cluster 2 isolates further differentiated into three subgroups. Cluster 1 (from AAUH_7CCFec@ to AAUH_12CDo) contained 52 GS1 isolates and the reference genome ATCC 33237. Cluster 2 subgroup 1 (from AAUH_44sig@ to AAUH_20HCsig@) contained 14 GS2 isolates and the reference genome 13826. Cluster 2 subgroup 2 (from AAUH_31CCRec1@ to AAUH_40UCf) contained 50 GS2 isolates. Cluster 2 subgroup 3 (from AAUH_27CCSigP3@ to AAUH_16UCdp5) contained 64 GS2 isolates. Branches indicate origin of the isolates: MC (blue) and isolates from Kirk et al. [18] and the reference genomes (yellow). [file 13099_2020_397_MOESM2_ESM.tif]
